# Supplementary figures and images for: Comparative genomic insight into the myxobacterial carbohydrate-degrading potential
Source: Front Microbiol. 2025 May 7;16:1550287. doi: 10.3389/fmicb.2025.1550287 (PMC12093494; doi:10.3389/fmicb.2025.1550287)

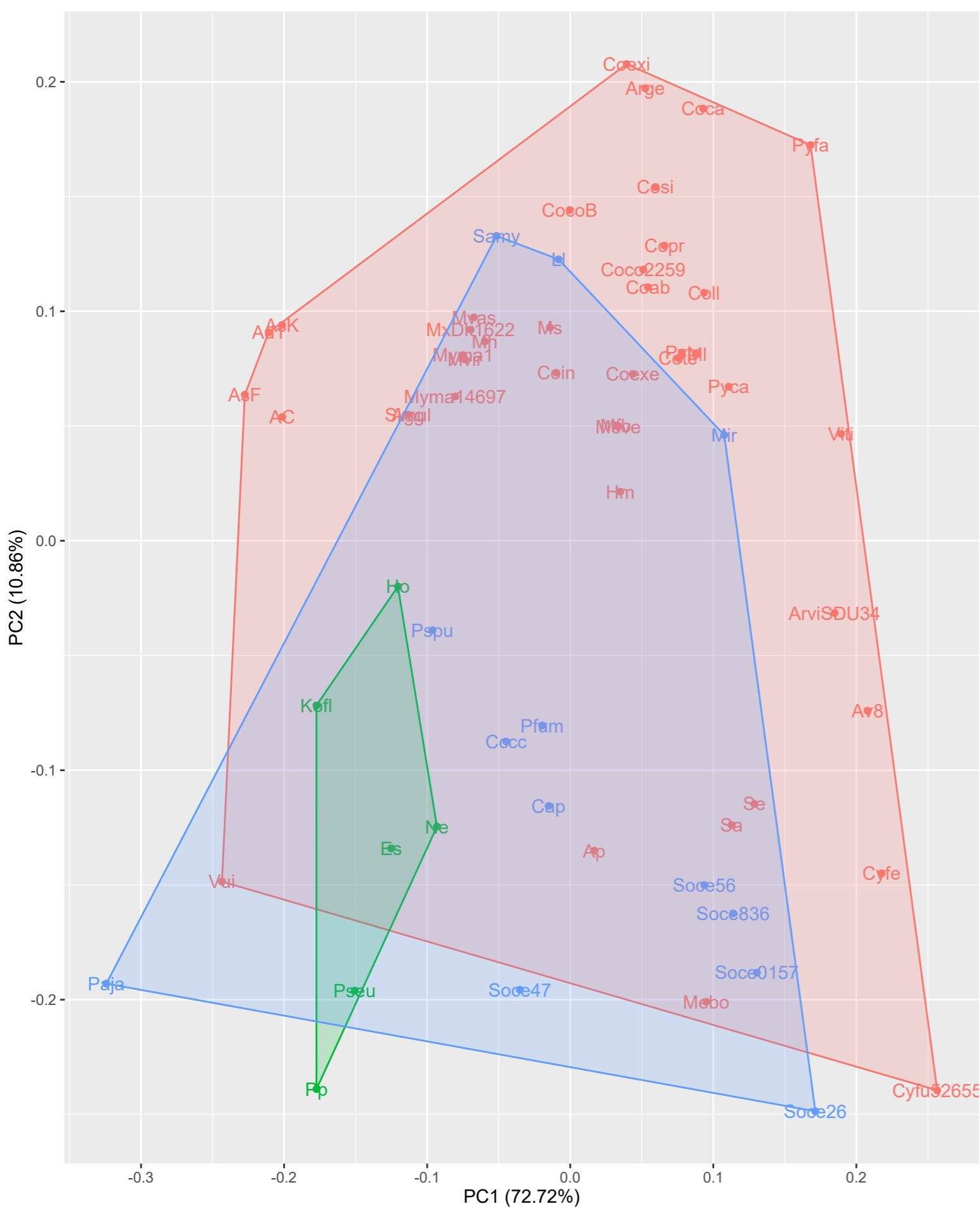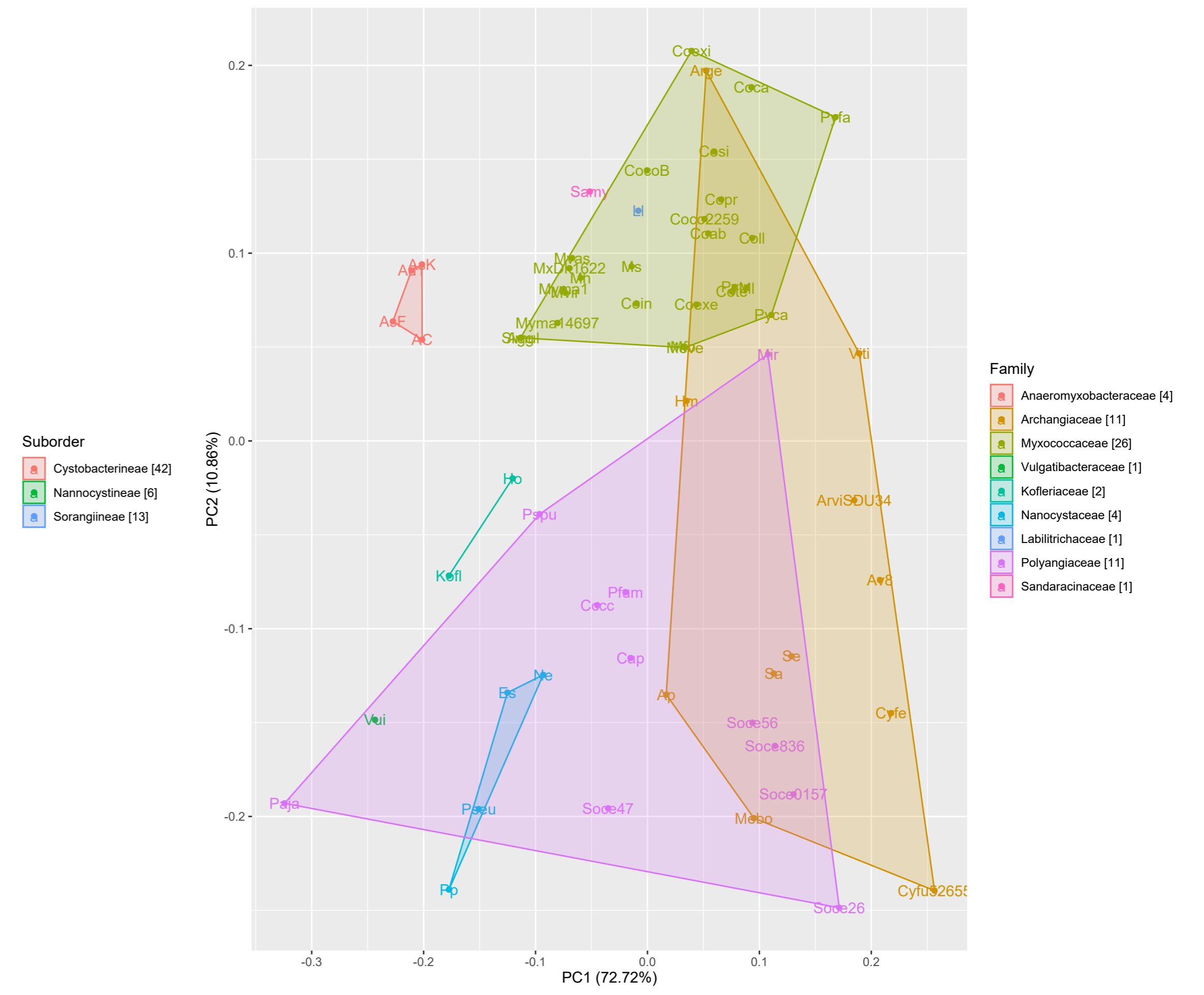

Supplement: SUPPLEMENTARY FIGURE 1 — Principal component analysis based on the organism-wise distribution of the six CAZyme Categories—GH, GT, AA, PL, CE, CBM—showing (a) suborder & (b) family-wise clustering. The numbers mentioned in brackets in the legend and X-axis depict the number of organisms in that suborder/family. [file Image_1.pdf]
